# Risk Factors of Persistent Pulmonary Hypertension in Neonate in A Tertiary Care Referral Center

**DOI:** 10.7759/cureus.22416

**Published:** 2022-02-20

**Authors:** Essam I Jastania, Mohammed S Alqarni, Abdulkarim W Abukhodair, Ziad M Bukhari, Rima A Bukhari, Sawsan Khatrawi, Norah Alsomali, Rahaf Waggass

**Affiliations:** 1 College of Medicine, King Abdullah International Medical Research Center, King Saud Bin Abdulaziz University for Health Sciences, Jeddah, SAU; 2 Cardiology, King Faisal Cardiac Center, King Abdulaziz Medical City, Jeddah, SAU

**Keywords:** neonatal intensive care unit, mortality rate, risk factors, pulmonary circulation, persistent pulmonary hypertension of the newborn

## Abstract

Background: Persistent pulmonary hypertension of the newborn (PPHN) is a condition in which pulmonary vascular resistance fails to decrease after birth. PPHN leads to hypoxemia due to right-to-left shunting of the blood through the fetal circulation. This study aimed to determine the association between PPHN and prematurity in neonates admitted to the neonatal intensive care unit (NICU).

Materials and methods: This study is a single-center, retrospective, and cross-sectional study. Patients diagnosed with PPHN had been selected by using a non-probability consecutive sampling technique from 2016 to 2020 at King Abdulaziz Medical City in Jeddah, Saudi Arabia. Patients with PPHN who did not admit to NICU were excluded.

Results: Fifty-six patients had met the inclusion and exclusion criteria. Twenty-six neonates were born prematurely before 37 weeks of gestation, and 30 were born at 37 weeks or more. Among the study population, respiratory complications were seen in 30 patients with a rate of 53.6%. The most common complications were respiratory failure, persistent pulmonary hypertension, and cardiopulmonary arrest.

Conclusion: Mortality was documented in 26 patients, with the complicated group having a rate of 73.3% compared to the uncomplicated group 15.4%. The most common complications seen in our patients were respiratory failure, persistent pulmonary hypertension, and cardiopulmonary arrest.

## Introduction

Persistent pulmonary hypertension of the newborn (PPHN) is a condition in which pulmonary vascular resistance fails to decrease after birth [1]. PPHN leads to hypoxemia due to right-to-left shunting of the blood through the persistent fetal circulation such as ductus arteriosus or foramen ovale [2]. Its prevalence was estimated to be approximately 1.9 per 1000 live births in a state-wide multi-center cohort study published in 2000 [3]. Some factors associated with PPHN include perinatal asphyxia, infections, meconium aspiration syndrome, and early gestational age [4]. A 2015 study strongly associated early gestational age with a worse neonatal outcome; it found severe PPHN requiring extracorporeal membrane oxygenation (ECMO) to be more prevalent in late preterm (34-37 weeks) and early term (37-39 weeks) infants [5].

PPHN is associated with developmental lung disorders, including but not limited to alveolar capillary dysplasia, genetic abnormalities of surfactant production, lung hypoplasia, and diaphragmatic hernias [5]. It was also found that, by 18 months of age, PPHN caused a neurodevelopmental delay, cerebral palsy, hearing deficits, and blindness [6]. Moreover, a recent study in California in 2019 looked at hospital records from 2005-2012 and concluded that post-discharge morbidity is high. The readmission rate was 28.6% and 9% during the first year of life in infants with and without PPHN, respectively. As for the mortality rate, it was higher at discharge (6.5%) compared to one-year post-discharge (0.7%) [7].

This study aimed to determine the association between PPHN and prematurity in neonates admitted to the neonatal intensive care unit (NICU) at King Abdulaziz Medical City (KAMC) from January 2016 to January 2020. Our secondary objectives were to identify the risk factors and determine the incidence of PPHN.

## Materials and methods

This study is a single-center, retrospective, and cross-sectional study. Patients diagnosed with PPHN had been selected by using a non-probability consecutive sampling technique from 2016 to 2020 at King Abdulaziz Medical City in Jeddah, Saudi Arabia. The study was ethically approved by the Institutional Review Board (IRB) at King Abdullah International Medical Research Center (KAIMARC), reference number IRBC/1203/21. The inclusion criteria were all patients diagnosed with PPHN and admitted to the NICU from 2016 to 2020. Based on echocardiography, PPHN was diagnosed as a chronically elevated mean pulmonary arterial pressure (mPAP) of ≥ 20 mm Hg at rest with increased pulmonary vascular resistance. Patients with PPHN who did not admit to NICU were excluded. This research commenced by reviewing the inclusion and exclusion criteria on the BestCare system (Riyadh, Saudi Arabia: SKHIC), and a total of 56 patients were identified. A datasheet was constructed which had demographic variables such as gender, weeks of gestations, height, weight, type of delivery, and appearance, pulse, grimace, activity, and respiration (Apgar) score, clinical manifestations such as hypoxia, respiratory distress, pneumothorax, pulmonary hypoplasia, and cardiac findings such as mitral regurgitation, ventral septal defect, myocardial dysfunction, presence of patent ductus arteriosus, foramen ovale, right ventricular hypertrophy, and right atrial hypertrophy. Other variables from the treatment regimen that were recorded include the use of Sildenafil, inotropes, vasodilators, surfactant, nitric oxide, fractional inspired oxygen (FiO2), high-frequency ventilation (HFV), and ECMO. Patients' outcomes were also collected in the datasheet, including length of stay, readmission, respiratory complications, and mortality.

The data analysis for this study was generated using IBM SPSS version 23 (Chicago, IL: IBM Corp.). For the continuous variables, the mean (standard deviation) and median (interquartile range) were reported for normally distributed data and skewed data, respectively. For the categorical variables, frequency and percentages were used. Two-tailed Student's t-test was used to compare means and chi-square test was performed for comparisons of categorical variables. A P-value of <0.05 was considered statistically significant.

## Results

Fifty-six patients had met the inclusion and exclusion criteria. The study population had a gestational age ranging from 23 to 24 weeks with a mean of 25±6 weeks. Twenty-six neonates were born prematurely before 37 weeks of gestation, and 30 were born at 37 weeks or more. Thirty-eight patients (67.9%) were males and 18 (32.1%) were females. Among the study population, respiratory complications were seen in 30 patients with a rate of 53.6%. The most common complications were respiratory failure (n=8), persistent pulmonary hypertension (n=7), and cardiopulmonary arrest (n=5). Details about the different types of respiratory complications are listed in Table 1.

**Table 1:** Types of respiratory complications among the study population.

Type of complication	n (%)
Respiratory failure	8 (14.3%)
Pulmonary hemorrhage	3 (5.4%)
Cardiopulmonary arrest	5 (8.9%)
Chronic lung disease	3 (5.4%)
Pulmonary edema	1 (1.8%)
Chylothorax	1 (1.8%)
Pleural effusion	1 (1.8%)
Persistent pulmonary hypertension	7 (12.5%)
Pulmonary interstitial emphysema	1 (1.8%)
No complications	26 (46.4%)

Mean one-minute and five-minute Apgar scores were four and seven in the respiratory complication group, respectively, compared to six and eight in the uncomplicated group. However, there was no statistically significant difference. For the gestational weeks, the complication group had a mean of 33.5 weeks compared to the uncomplicated group, which had a mean age of 36 weeks. Gender of the neonates, type of delivery, and diabetes during pregnancy had equal rates among the complicated and uncomplicated patients. For the clinical presentation of the study population, 40 patients (71.4%) had hypoxia, and 27 (48.2%) of the hypoxic patients had it during the 24 hours after birth with a mean oxygen saturation of 89±11%. Also, metabolic acidosis was seen in 18 (32.1%) patients, respiratory distress in 37 (66.1%) patients, meconium aspiration syndrome in nine (16.1%) patients, pneumonia in 12 (21.4%) patients, pulmonary hypoplasia in eight (14.3%) patients, and pneumothorax in 15 (26.8%) patients. PPHN and prematurity in neonates admitted to the neonatal intensive care unit (NICU) show no statistical significance. Cardiac findings after examination of the study population are listed in Table 2.

**Table 2:** Cardiac findings of the study population.

Variable		n (%)
Myocardial dysfunction	Yes	4 (7.1%)
	No	52 (92.2%)
Loud second heart sound	Yes	3 (5.4%)
	No	53 (94.6%)
Shunt across the foramen ovale	Left to right shunt	5 (8.9%)
	Right to left shunt	2 (3.6%)
	Bidirectional	4 (7.1%)
	No shunt	45 (80.4%)
Shunt across the ductus arteriosus	Left to right shunt	6 (10.7%)
	Right to left shunt	1 (1.8%)
	Bidirectional	2 (3.6%)
	No shunt	47 (83.9%)
VSD	Yes	15 (26.8%)
	No	41 (73.2%)
Mitral regurgitation	Yes	3 (5.4%)
	No	53 (94.6%)
Tricuspid regurgitation	Yes	6 (10.7%)
	No	50 (89.3%)
Right ventricular hypertrophy	Yes	6 (10.7%)
	No	50 (89.3%)

Down syndrome was diagnosed in four (7.1%) of the neonates. Table 3 shows the different therapies used among the two study groups. Mortality was documented in 26 patients, with the complicated group having a mortality rate of 73.3% compared to the uncomplicated group of 15.4% (p-value <0.001).

**Table 3:** Medications and therapies used for the respiratory complicated and uncomplicated groups. *chi-square test was used

Variable		Overall n=56	Complicated group n=30	Uncomplicated group n=26	p-Value*
Sildenafil	Yes	18	6 (33.3%)	12 (66.7%)	0.03
	No	38	24 (63.2%)	14 (36.8%)	
Inotropes	Yes	38	24 (63.2%)	14 (36.8%)	0.03
	No	18	6 (33.3%)	12 (66.7%)	
Intravenous vasodilator	Yes	7	2 (28.6%)	5 (71.4%)	0.2
	No	49	28 (57.1%)	21 (42.9%)	
Surfactant	Yes	20	15 (75%)	5 (25%)	0.01
	No	36	15 (41.7%)	21 (58.3%)	
Nitric oxide	Yes	27	17 (63%)	10 (37%)	0.1
	No	29	13 (44.8%)	16 (55.2%)	
High-frequency ventilation	Yes	28	18 (64.3%)	10 (35.7%)	0.1
	No	28	12 (42.9%)	16 (57.1%)	

## Discussion

According to the clinical characteristics of PPHN associated with Down syndrome, 17 infants with Down syndrome (DS) did not have any structural, congenital heart diseases (CHD) but presented with PPHN. It was noticed that pulmonary hypertension (PH) was resolved in most patients, while two infants that had refractory PH benefited from patent ductus arteriosus (PDA) ligation [8]. An autopsy was performed on two other infants and showed findings of immature lung cells, thus deducting that there’s a risk of PH in DS infants even without structural heart diseases [8]. The patients with DS and PPHN were reviewed retrospectively from a hospital database in Toronto that admitted sick children with pulmonary hypertension between 1988 and 2001 in the neonatal, cardiology department. DS was diagnosed in infants based on clinical evaluation and confirmed via chromosomal analysis [8]. The criteria used to diagnose PPHN were achieved through hypoxemia refractory to oxygen therapy or lung recruitment strategies and not having any clear findings of pulmonary parenchymal diseases, only signs of directional right to left shunting can be present at a ductal or arterial level [8]. According to the results, there was a correlation and significant proportion of DS infants that had PH in the absence of CHD, and suggestions that hypoxemia in admitted DS infants should be evaluated for any presence of structural CHD. Patients that did not show signs of structural CHD should have the possibility of PPHN considered. Thus, it is recommended to continue surveillance and close the open ducts after PH resolution [8]. Regarding the mortality in our patients compared to the findings in this study, we found that out of our 26 patients, the documented mortality rate was around 15.4% in uncomplicated cases.

A study was conducted to measure the prevalence in Down syndrome patients that had persistent pulmonary hypertension and congenital heart defects under the Dutch Pediatric Surveillance Unit aimed to find a correlation between CHD and PPHN with DS. It was shown that when compared to the general population, in neonatal patients with DS there was a significant 43% prevalence of CHD, while having a marked increased elevation in the incidence of PPHN (5.2%) [9]. There were no direct or indirect correlations between CHD and factors relating to the DS children during their neonatal periods such as Apgar score, gestational age, and birth weight. It was shown that some patients were not the trisomy 21, nor had complete data on any cardiac involvement. An obstacle was faced during the conduction of this study; during the few weeks after birth in some children with DS, there were no signs of major cardiac malformations nor any associations with pulmonary hypertension [10]. In DS patients that underwent marked and irreversible damage to the pulmonary vasculature, further exclusion of CHD can’t simply be made using a normal neonatal examination [10-12].

The conducted studies and results follow our shared goals and objectives of the importance of detecting structural heart defects during the neonatal periods in DS patients, with appropriate methods such as echocardiography. The importance of early detection is well supported by the DS health care guidelines published by the American Academy of Pediatrics and the Down Syndrome Medical Interest Group of the United Kingdom and Ireland [13]. Early diagnosis and management are the keys to avoiding irreversible hemodynamic consequences of the defect. Regarding CHD, it was shown in a conducted study aimed at finding the prevalence of structural heart diseases in neonates with DS and also persistent pulmonary hypertension that it is very common [13]. Ventricular septal defects (VSD) are the most common cardiac defect, and atrioventricular septal defect (AVSD) is second to VSD [13]. Out of the 316 patients diagnosed with severe CHD, VSD was the most commonly diagnosed lesion (22.5%) and did not show any significance regarding mortality or complications [12]. Compared to our study, many of our patients had VSDs 15 (26.8%), while others were around 41 (73.2%) that did not have congenital heart disease. The patients frequently develop pulmonary hypertension at a younger age before one year. A study was conducted at Shahid Gangalal National Heart Centre many cardiac patients came or were transferred; however, they could not reflect the nation's results [1]. Many cases were underdiagnosed or missed due to not having access to healthcare services from their remote hometowns, low socioeconomic status, or death prior to the diagnosis [1].

Limitations

This retrospective study with a single-center design had a limited sample size which affected generalizability. It has an interpretive bias due to lack of information of our patients during the study duration. Also, an inherent selection bias because it was retrospective. Many patients were excluded due to insufficient data and information.

## Conclusions

PPHN is considered a significant source of postnatal morbidity and mortality. Mortality was documented in 26 patients, with the complicated group having a rate of 73.3% compared to the uncomplicated group with a rate of 15.4%. That emphasizes the importance of recognizing these complications early to improve the outcome and reduce the mortality rate. The most common complications seen in our patients were respiratory failure, persistent pulmonary hypertension, and cardiopulmonary arrest. Apgar scores, type of delivery, gender of the neonates, and diabetes during pregnancy were not statistically significant. Cardiac screening by echocardiography is crucial. Early diagnosis and management are the keys to avoiding irreversible hemodynamic consequences of the defect. The inability to treat PPHN increases the risk for postnatal complications which extremely affect the outcomes of these infants.
